# USP13 Suppresses Colorectal Cancer Angiogenesis by Downregulating VEGFA Expression through Inhibition of the PTEN-AKT Pathway

**DOI:** 10.32604/or.2025.060440

**Published:** 2025-07-18

**Authors:** Guo-Zhi Xu, Han-Yang Guan, Yan-Guan Guo, Yi-Ran Zhang, Jing-Hua Pan, Simin Luo, Hui Ding, Yunlong Pan, Qi Yao

**Affiliations:** 1 Department of Gastrointestinal Surgery, The First Affiliated Hospital of Jinan University, Guangzhou, 510632, China; 2 Department of Hepatobiliary Gastrointestinal Surgery, Xinyi People’s Hospital, Maoming, Xinyi, 525300, China; 3 Department of Bone and Joint Surgery, The First Affiliated Hospital, Jinan University, Guangzhou, 510632, China; 4 Department of Anorectal Surgery, The Second Clinical Medical College, Jinan University (Shenzhen People’s Hospital), Shenzhen, 518000, China

**Keywords:** Colorectal cancer (CRC), angiogenesis, ubiquitin-specific peptidase 13 (USP13), Vascular Endothelial Growth Factor A (VEGFA)

## Abstract

**Background:**

Tumor angiogenesis is related to solid tumor occurrence. Ubiquitin-specific peptidase 13 (USP13) is a deubiquitinating enzyme with a pivotal effect on tumor proliferation, metastasis, and tumorigenesis. Nonetheless, its effect on colorectal cancer (CRC) angiogenesis remains poorly understood.

**Methods:**

Human umbilical vein endothelial cells (HUVECs) and CRC cells were cultivated, followed by USP13 knockdown/overexpression using shRNA lentiviral vectors or plasmids. Conditioned media (CM) from treated CRC cells were collected to assess HUVEC migration, invasion, and tube formation. Phosphatase and tensin homolog (PTEN) overexpression and recombinant vascular endothelial growth factor A (VEGFA) rescue experiments were performed. Molecular mechanisms were analyzed via Western blot (PTEN, p-AKT, VEGFA), co-immunoprecipitation (PTEN ubiquitination), and *in vivo* nude mice study to detect the role of USP13 in tumor angiogenesis.

**Results:**

USP13 expression in CRC cells is downregulated and negatively related to platelet endothelial cell adhesion molecule-1 (CD31) expression. Furthermore, the conditioned medium from CRC cells with USP13 knockdown significantly promoted HUVEC migration, invasion, and tube formation, while USP13 overexpression exerted the opposite effect. Additionally, USP13 overexpression significantly increased PTEN expression while decreasing protein kinase B (AKT) phosphorylation levels. Concurrently, USP13 overexpression significantly reduced PTEN ubiquitination, whereas USP13 knockdown remarkably increased this modification. Overexpression of PTEN in sh-USP13 CRC cells decreased the expression levels of VEGFA and p-AKT. USP13 also inhibited tumor angiogenesis through downregulating VEGFA, and recombinant VEGFA blocked the inhibition of the conditioned medium from USP13-overexpressing CRC cells against HUVEC angiogenesis *in vivo*.

**Conclusions:**

USP13 downregulated VEGFA and inhibited tumor angiogenesis via the PTEN-AKT pathway.

## 1 Introduction

Colorectal cancer (CRC) ranks third among cancers globally, and it accounts for the third factor inducing cancer-associated death [1]. Recent advancements in targeted therapy, immunotherapy, radiotherapy, and chemotherapy have substantially increased 5-year survival rate in CRC cases [2]. However, tumor metastasis and recurrence remain challenging obstacles in the clinical management of CRC. Consequently, it is urgently needed to elucidate molecular mechanisms of the CRC pathogenic mechanism and obtain new anti-CRC therapeutic targets.

Angiogenesis is the critical phase in the development of solid cancers and an important hallmark of malignancy [3–5]. Accumulating evidence demonstrates that angiogenesis provides essential nutrients and oxygen for cancer cell survival, thereby exerting an important effect on tumor occurrence, particularly in CRC cell growth and migration [6,7]. Antiangiogenic therapy, in line with the “tumor starvation” concept, emerges as a promising strategy for treating various human malignant tumors, including CRC [8]. The U.S. Food and Drug Administration approved antiangiogenic medications targeting vascular endothelial growth factor (VEGF), like bevacizumab, for use as first-line therapy for CRC patients [9]. Numerous studies have revealed that multiple genes abnormally expressed in cancer cells stimulate and recruit endothelial cells to promote vascular development [10]. In CRC cells, *PELP1* gene knockdown markedly reduces angiogenesis by suppressing the STAT3/VEGFA pathway [3]. However, the molecular mechanisms of angiogenesis remain to be fully elucidated.

Ubiquitin-specific peptidase 13 (USP13) is a member of the deubiquitinase (DUB) superfamily. Although previous research has indicated that USP13 may influence cancer growth by regulating the stability of oncogenes and tumor suppressor proteins, its role in various malignancies remains inconsistent. For instance, USP13 is responsible for deubiquitinating and stabilizing microphthalmia-associated transcription factor, an important regulatory factor for human melanoma, which is unique to its spectrum of activity [11]. USP13 also facilitates glioblastoma and ovarian cancer occurrence through stabilizing c-MYC and ACLY/OGDH [12]. Furthermore, USP13 plays a role in critical cellular processes, particularly in carcinogenesis, by modulating tumor suppressors such as p53 and phosphatase and tensin homolog (PTEN) [13]. Additionally, USP13 stabilizes WISP1 through deubiquitination, thereby enhancing esophageal squamous cell carcinoma development by activating the Wnt/CTNNB1 pathway [14]. USP13 also promotes the proliferation and autophagy of acute myeloid leukemia cells by enhancing ATG5 activity [15]. In contrast, USP13 inhibits the progression of bladder cancer by enhancing PTEN expression, which is regulated by NF-κB [16]. Based on the above results, USP13 has an important effect on tumor angiogenesis. Therefore, its effect on CRC angiogenesis warrants further investigation.

PTEN is a known tumor suppressor with negative regulation of the AKT pathway [17]. It functions through the dephosphorylation of phosphatidylinositol (3, 4, 5)-trisphosphate into phosphatidylinositol (4, 5)-bisphosphate, thus limiting AKT activation [17]. Current PTEN regulation mechanisms include loss or inhibition of PTEN expression due to mutations in the *PTEN* gene or chromosomal deletions, which can result in loss of function, abnormal post-translational modifications, activation of oncogenic pathways, and dysregulation of noncoding RNAs [17]. This work analyzed the relationship of microvessel density (MVD) with USP13 levels among 40 patients with CRC. By conducting *in vitro* and *in vivo* functional analyses, USP13 significantly inhibited CRC angiogenesis by reducing the ubiquitination of PTEN and modulating the PTEN-AKT-VEGFA pathway.

## 2 Materials and Methods

### 2.1 Clinical Samples and Patients

Forty CRC and 40 matched non-carcinoma tissue samples were collected from patients from the First Affiliated Hospital of Jinan University (Guangzhou, China), and subjected to histopathological and clinical examination. The study protocol gained approval from the Institutional Research Ethics Committee of Jinan University (JNUKY-2023-0062) and was carried out following the Declaration of Helsinki. Informed consent was obtained from patients.

### 2.2 Cell Culture

HCT116 and HT29 cells were cultivated within DMEM (Gibco, #11965, Shanghai, China) that contained 10% fetal bovine serum (FBS, Biological Industries, #04-001-1A, Shanghai, China) as well as 1% penicillin-streptomycin (Sangon Biotech, #E607011, Shanghai, China) under 37°C and 5% CO_2_ conditions. Human umbilical vein endothelial cells (HUVECs), provided by the Institute of Precision Oncology Medicine and Pathology, School of Medicine, Jinan University, were cultured in ECM medium (Solarbio, #YZ-1001, Beijing, China) that contained 10% FBS and 1% penicillin-streptomycin under 5% CO_2_ and 37°C conditions. All cells used in this study were free of mycoplasma contamination.

### 2.3 Immunohistochemistry (IHC) Assay

IHC assay was conducted on 4-µm-thick, formalin-fixed, paraffin-embedded tissue sections, which underwent xylene deparaffinization, gradient ethanol rehydration, and membrane permeabilization through incubation with 0.1% Triton X-100 in PBS for 10 min at room temperature. Antigen retrieval was subsequently performed through heat-induced epitope retrieval in citrate buffer (pH 6.0) for 15 min. After cooling, sections were incubated using 3% hydrogen peroxide (H_2_O_2_) contained in methanol at room temperature for a 15 min duration to quench endogenous peroxidase activity. The 5% normal serum was added to block sections for reducing nonspecific binding, followed by overnight section incubation under 4°C using primary antibodies: anti-USP13 antibodies (Santa Cruz Biotechnology, Dallas, TX, USA; #SC144160; 1:200), anti-human cell adhesion molecule-1 (CD31) antibodies (Servicebio, Wuhan, China; #GB113151; 1:500), and anti-human VEGFA antibodies (Proteintech, Wuhan, China; #19003–1-AP; 1:1000). Subsequently, the sections were incubated with biotinylated secondary antibodies (1:500) (Abcam, Goat Anti-Rabbit and Anti-Mouse IgG H&L (Biotin, ab6720 and ab6788, Shanghai, China) under ambient temperature for a 1-h duration, followed by staining with an avidin-biotin complex. Visualization was performed using diaminobenzidine (Sigma, D12384, Shanghai, China), which yielded a brown precipitate. Hematoxylin counterstaining (Sigma, H9627, Shanghai, China) was completed to enhance cellular contrast. The light microscope (Mshot, MF52-N, Guangzhou, China) was adopted to examine these sections, and image analysis was conducted using ImageJ (version 1.46, National Institutes of Health, Bethesda, MD, USA) for quantifying positive staining intensity and positive cell percentage. Statistical analyses were implemented to evaluate whether the positively stained sections were of significance, which contributed to our understanding of the expression patterns of target antigens in cancerous tissues.

The IHC staining pattern was scored to assess protein expression in tissue samples, primarily for detecting tumor markers. This scoring system comprises two primary components: staining intensity and positive cell proportion. The staining intensity is rated by a scale from 0 (non-staining) to 3 (strong staining), whereas positive cell percentage is rated from 0 (0% positive) to 4 (76%–100% positive). The IHC score is derived by multiplying these two values, yielding a score typically ranging from 0 to 12. To categorize high and low expression of USP13 in this study, we established the following threshold for IHC scores: scores ≥ 6, high expression; scores < 6, low expression.

### 2.4 Cell Transfection and shRNA

By specific protocols, cells were subjected to either VEGFA siRNA (Thermo Fisher Scientific, 284703, Shanghai, China), PTEN siRNA (Cell Signaling Technology, #6251, Shanghai, China) or control siRNA (5^′^-UUCUCCGAACGUGUCACGUdTdT-3^′^) transfection by using Lipofectamine 2000 (Thermo Fisher Scientific, 11668030, Shanghai, China). The shRNA lentiviral vector pLKO.1 targeting USP13 was constructed by utilizing the target sequence: 5^′^-CGATTTAAATAGCGACGATTA-3^′^. For the negative control, a vector containing scrambled shRNA was obtained from Addgene (USA). USP13 overexpression was achieved by transfecting cells with a plasmid encoding the full-length human USP13 cDNA (pCMV-USP13, Sino Biological, HG10001-UT). Briefly, cells (2 × 10^5^/well) were inoculated into 6-well plates and transfected with Lipofectamine 3000 (Thermo Fisher, L3000015) following specific instructions. At 48 h later, transfected cells were subjected to selection using 500 μg/mL hygromycin B (Sigma, H0654) for 7 days. Overexpression efficiency was confirmed via Western blot using an anti-USP13 antibody (Proteintech, 14617-1-AP). Control cells were transfected with an empty vector (pCMV-3 × Flag, Sigma, E7033) under identical conditions. Subsequently, real-time quantitative polymerase chain reaction (RT-qPCR) and Western blotting (WB) assay were conducted to validate transfection efficacy.

### 2.5 Conditioned Medium (CM)

CRC cells (1.0 × 10^6^) were cultivated overnight into 6-well plates; subsequently, 2 mL of DMEM was added to every well. The CM was obtained 24 h later, followed by 15 min of centrifugation at 1500× *g* and storage under −80°C. This CM was utilized in enzyme-linked immunosorbent assay (ELISA), Transwell, and tube formation assays.

### 2.6 Transwell Migration and Invasion Assays

Transwell assays were conducted according to prior depiction [18]. Migration and invasion assays using HUVECs were carried out in a coated Transwell chamber (Corning, New York, NY, USA; #353097). HUVECs (1 × 10^3^) were seeded onto the top chamber (Corning; #354248, Shanghai, China) with or without the Matrigel matrix (Corning, #354230, Shanghai, China). The bottom chamber contained 200 µL basal ECM and 800 µL CM (with/without recombinant VEGFA [rVEGFA]). These chambers were subjected to 36 h of incubation under 37°C using 5% CO_2_. Following fixation, the cells onto top membrane were eliminated using a cotton swab. Membrane-bound cells were then immobilized before 10 min of 0.1% crystal violet staining at room temperature. The light microscope (Mshot, MF52-N) was employed for image capturing, whereas ImageJ (version 1.46; National Institutes of Health) was adopted for quantifying invading cells in three random fields from each well.

### 2.7 Tube Formation Assay

HUVECs (cell density: 1.0 × 10^4^/well) were inoculated on the 96-well plate covered with 50 µL of Matrigel matrix and later cultivated with CM under 37°C and 5% CO_2_ conditions for 4 h. A microscope (Mshot, MF52-N) was used to capture four random images, while ImageJ software (version 1.46) was applied to the analysis.

### 2.8 ELISA

ELISA kits (NeoBioscience, Shenzhen, China; #EHC108 for VEGFA) were utilized to quantify protein levels of VEGFA in cell culture supernatants in accordance with the manufacturer’s instructions.

### 2.9 RNA Extraction and Real-Time PCR

The RNA extraction kit (Solarbio; #R1200) was utilized to extract total RNA following specific protocols. To conduct single gene analysis, total RNA (1 μg) was used to prepare cDNA by PrimeScript RT Master Mix (Takara, Shiga, Japan; #RR036A) with the 20 μL reaction system for a 15-min duration under 37°C and for a 5-s duration under 85°C. PCR analysis was conducted using a PCR system (Bio-Rad Thermal Cycler (Model: T100), Hercules, CA, USA) with SYBR Green Master Mix (Vazyme, Nanjing, China; #Q121-02-AA) in CFX96 Touch™ real-time PCR system (Bio-Rad Thermal Cycler, CA, USA) following specific protocols. The cycling procedure was shown below: one cycle under 95°C for 5 min; 10 s of amplification under 95°C and 30 s under 60°C for 40 cycles. The target mRNAs included VEGFA, VEGFB, VEGFC, VEGFD, PDGFB, PDGFC, THBS1, WNT7B, PGF, bFGF, MMP2, and CXCL9; the ACTB mRNA was an endogenous reference. The primers utilized in RT-qPCR can be observed from Table A1.

### 2.10 Western-Blot

Protein extracts were prepared from CRC cells transfected with USP13-specific shRNA or overexpression plasmids with RIPA lysis buffer (Beyotime, P0013B, Shanghai, China) that contained protease/phosphatase inhibitors (Roche, 04906837001, Shanghai, China). Protein content was analyzed via Bicinchoninic Acid (BCA, Thermo Fisher Scientific, A55864, Shanghai, China) assay. Protein aliquots (30 μg/lane) were subjected to 10% SDS-PAGE for separation before transfer onto Poly(vinylidene fluoride) (PVDF) membranes (Millipore, IPVH00010, Shanghai, China). 5% defatted milk contained in Tris-buffered saline Tween (TBST) was added to block membranes under ambient temperature for a 1-h duration, followed by overnight incubation using primary antibodies under 4°C: anti-USP13 (1:1000, Abcam, #ab109264), anti-PTEN (1:1000, CST, #9552), anti-AKT (1:2000, CST, #9272), anti-phospho-AKT (Ser473, 1:1000, CST, #4060), anti-VEGFA (1:500, Santa Cruz, sc-53463), and anti-β-actin (1:5000, Sigma, A5441). Membranes were washed before a further 1 h of incubation using HRP-conjugated secondary antibodies (1:5000, CST) under ambient temperature. Protein band visualization was completed with ECL substrate (Millipore, WBULS0100, Shanghai, China) and quantified by ImageJ software.

### 2.11 PTEN Deubiquitylation In Vitro

For the *in vitro* PTEN deubiquitination experiment, HCT116 cells underwent transfection using Flag-PTEN, HA-ubiquitin, MYC-USP13, or control shRNA. Following transfection, the cells were exposed to 6 h of 10 µM MG132 (Thermo Fisher Scientific, J63250MCR, Shanghai, China) treatment. Flag-PTEN was then immunoprecipitated using Flag beads (MCE, HY-K0207, Shanghai, China) and subsequently immunoblotted with anti-HA and anti-Flag antibodies.

### 2.12 Animal Experiments In Vivo

A total of 16 Six-week-old female BALB/c mice were obtained in GemPharmatech Co., Ltd. (Nanjing, China) and housed in a specific pathogen-free (SPF) environment at the Experimental Animal Center of Jinan University. The mice were randomly assigned to distinct groups. HCT116 cells (1 × 10^7^) with either empty vector (EV) or USP13 overexpression were suspended in 200 μL PBS at a 1:1 ratio before subcutaneous injection in the right flank of mice. Measurements of tumor volume were conducted at 2-day intervals. For investigating whether inhibition of USP13 against angiogenesis is mediated by the PTEN pathway and VEGF, mice inoculated with EV-containing or USP13-overexpressing HCT116 cells were administered the PTEN-specific inhibitor SF1670 (MedChemExpress, #HY-15842, Shanghai, China) once every two days. Xenografts were collected after 3 weeks. The tumor tissue was paraffin-embedded and subjected to IHC assay. The animal experimental findings were acquired in a blinded manner. The animal experiments gained approval from the Institutional Animal Care and Use Committee of Jinan University (approval number: 20210720-010).

### 2.13 Statistical Analysis

GraphPad Prism 5.0 (La Jolla, CA, USA) was employed for statistical analysis. The mean values of the two groups were compared by Student’s *t*-test. Each experiment was conducted thrice. The *p*-value of < 0.05 stood for statistical significance.

## 3 Results

### 3.1 USP13 Up-Regulation in Tissue Samples of CRC Patients Negatively Correlates with MVD

**Figure 1:**
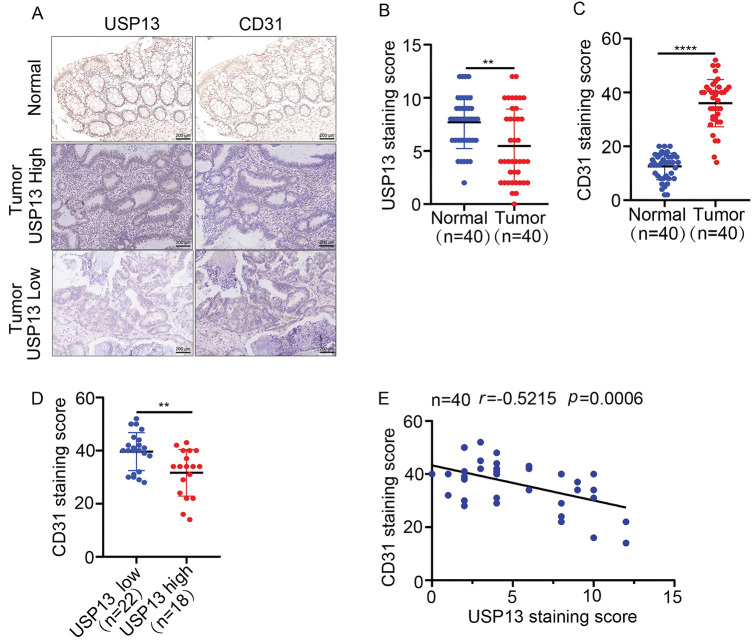
Analysis of the correlation between USP13 expression and CD31 level in tissues of CRC patients. (**A**) Images showing immunohistochemical staining of USP13 and CD31 of CRC and paired non-carcinoma tissues in 40 CRC patients. Scale bar: 50 μm. (**B, C**) Expression levels of USP13 (**B**) and CD31 (**C**) according to the staining indices among CRC tissue samples and paired non-carcinoma counterparts. (**D**) CD31 protein levels among patients with low or high USP13 expression. (**E**) Correlation analysis between the staining indices and USP13 and CD31 protein levels of human CRC samples (n = 40). The correlation coefficient (r) is shown. Results are represented by mean ± SEM (**p < 0.01, ****p < 0.0001)

For investigating the relation of USP13 expression with MVD of CRC and adjacent tissues, serial sections of tumor and adjacent tissues from 40 clinical CRC patients were subjected to IHC assay for USP13 and CD31 staining. As shown in Fig. 1A,B, CD31, a transmembrane glycoprotein, is a common endothelial marker of MVD and is primarily expressed by endothelial cells and various hematopoietic cells [10]. The IHC assay revealed a distinct difference in CD31 expression between CRC tumors and normal tissues (Fig. 1A,C). A subset of 22 cases with low USP13 expression and 18 cases with high USP13 expression was selected for further analysis. Tumor tissues from patients with high USP13 expression exhibited significantly lower CD31 expression as compared to those with low USP13 expression (Fig. 1D). Furthermore, USP13 expression was inversely related to CD31 expression in tissues of CRC patients (Fig. 1E). Therefore, USP13 has an important effect on modulating angiogenesis in CRC.

### 3.2 USP13 Suppresses Angiogenesis In Vitro

**Figure 2:**
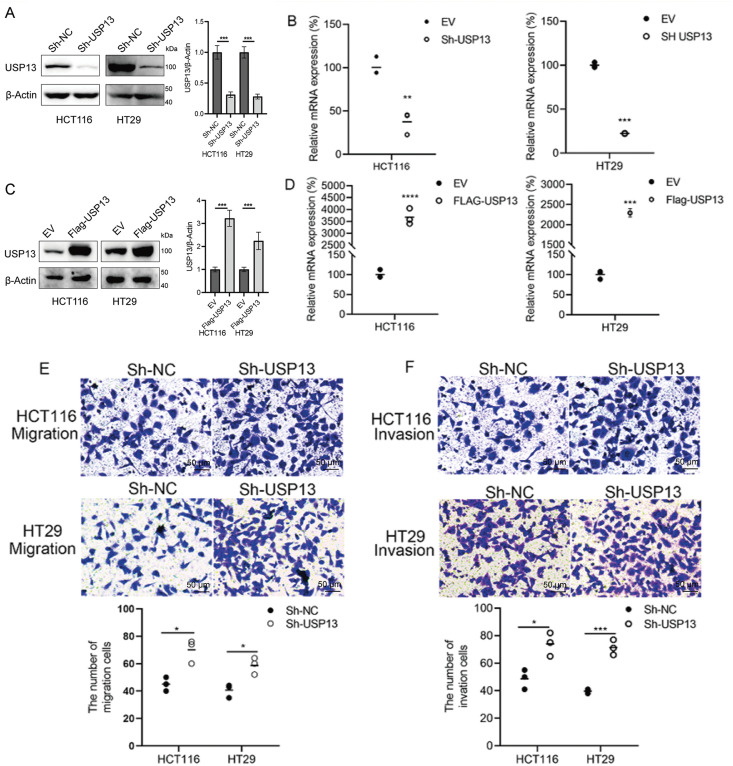

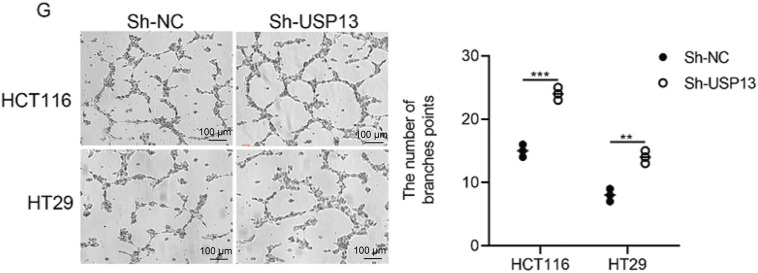
USP13 suppresses HUVEC migration, invasion, and tube formation abilities *in vitro*. (**A**) WB assay to determine USP13 levels of CRC stable cells with USP13 inhibition (sh-USP13) or the corresponding control cells (sh-NC). β-Actin was the loading control. (**B**) RT-qPCR-based detection of USP13 mRNA expression in HCT116 and HT29 stable cells with USP13 inhibition (sh-USP13) or the corresponding cells(sh-NC). (**C**) WB assay of CRC stable cell lines overexpressing USP13 (Flag-USP13) or control EV. β-Actin served as the loading control. (**D**) Detection of USP13 mRNA expression in HCT116 and HT29 stable cell lines overexpressing USP13 (Flag-USP13) or control EV. (**E, F**) Transwell assays were used to assess the cell migration (**E**) and invasion (**F**) of HUVECs after CM treatment from either sh-negative control (NC) or sh-USP13 cells. Scale bar: 50 µm. An image of three independent assays is presented. The bar graph displays the number of HUVECs that migrated to and invaded different regions. (**G**) Role of CM from sh-NC or sh-USP13 cells in the tube formation ability of HUVECs. Scale bar: 50 µm. The tube number formed is presented in a bar graph. A result from three independent assays is displayed. Data are represented by mean ± SEM. *p < 0.05; **p < 0.01; ***p < 0.001; ****p < 0.0001

**Figure 3:**
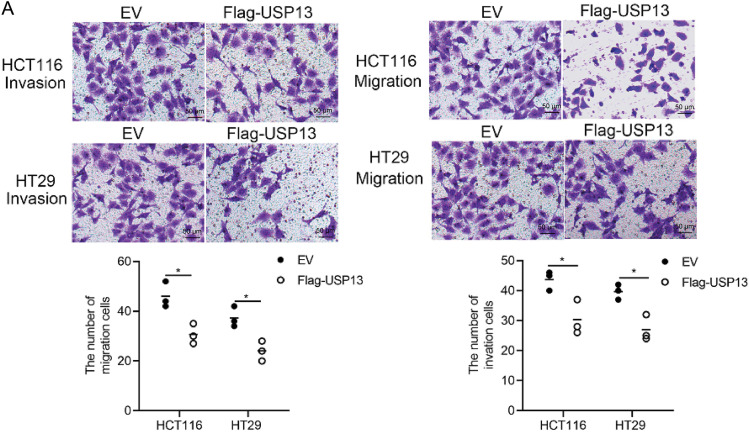

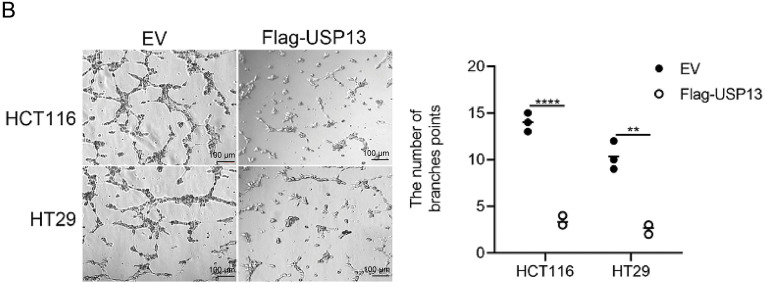
USP13 overexpression promotes angiogenesis of CRC. (**A**) Transwell assays were conducted to analyze HUVEC invasion and migration after CM treatment from Flag-USP13 cells or EV cells. The bar graph depicts HUVEC invasion and migration. (**B**) Role of CM from EV cells or Flag-USP13 cells in the tube formation of HUVECs. The bar graph displays the tube number formed. A result from three independent assays is displayed. Data are represented by mean ± SEM. *p < 0.05; **p < 0.01; ****p < 0.0001

For analyzing the role of USP13 in the angiogenesis of CRC tumors, we established stable knockdown cell lines, namely sh-USP13-HCT116 and sh-USP13-HT29, and stable overexpression cell lines, namely Flag-USP13-HCT116 and Flag-USP13-HT29, through lentiviral infection. The successful knockdown and overexpression of USP13 in CRC cell lines HCT116 and HT29 were confirmed by WB assay and qPCR analysis (Fig. 2A–D). Given that vascular endothelial cell migration and proliferation are essential processes of angiogenesis [19], we examined how USP13 affects HUVEC migration and proliferation. Notably, CM from sh-USP13 CRC cells markedly promoted HUVEC invasion and migration (Fig. 2E,F). We also assessed whether USP13 facilitates tube formation by HUVECs, which is crucial for all angiogenesis stages [20]. From Fig. 2G, CM in sh-USP13 CRC cells significantly increased the branch point number. Furthermore, stable USP13 overexpression in HCT116 and HT29 cells was utilized to complement loss-of-function studies. The HUVEC migration, invasion, and tube formation abilities were significantly decreased by CM from CRC cells overexpressing USP13 (Fig. 3A,B). Therefore, USP13 is important for HUVEC angiogenesis.

### 3.3 VEGFA Is Involved in the USP13-Mediated Reduction of Angiogenesis

**Figure 4:**
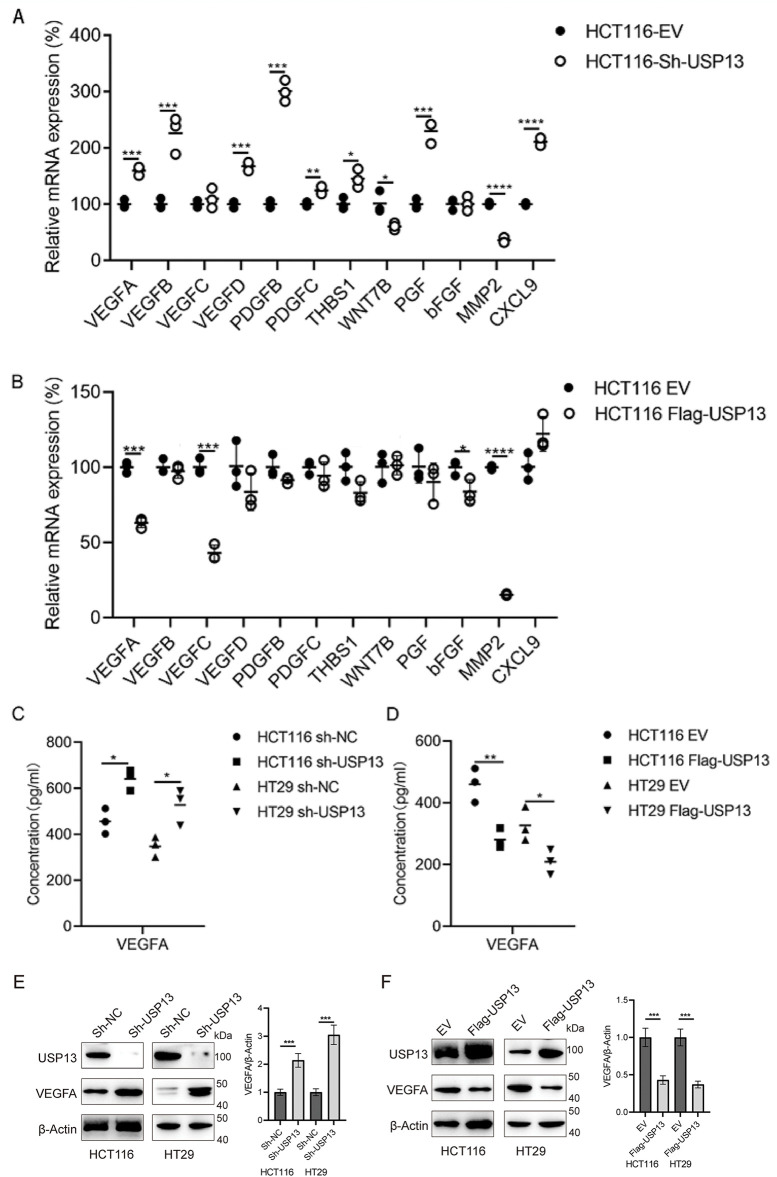
USP13 inhibits VEGFA expression in CRC cells. (**A, B**) RT-qPCR analysis of angiogenesis-related gene expression in sh-USP13 (**A**) and Flag-USP13 (**B**) HCT116 cells. (**C**) The secretion level of VEGFA in CM from sh-NC or sh-USP13 CRC cells was quantified through ELISA. (**D**) VEGFA protein level of CM from EV or Flag-USP13 CRC cells was quantified by ELISA. (**E**) WB assay of USP13 and VEGFA expression in sh-NC and sh-USP13 CRC cells, and β-actin was the loading control. (**F**) WB assay of USP13 and VEGFA expression in EV and Flag-USP13 CRC cells, and β-actin was the loading control. A result from three independent assays is displayed. Data are represented by mean ± SEM. *p < 0.05; **p < 0.01; ***p < 0.001; ****p < 0.0001

Given the involvement of several cytokines and growth factors related to tumor angiogenesis [21], this study investigated if USP13 affects the production of essential cytokines that promote angiogenesis. To test the above hypothesis, several angiogenesis-related cytokines were analyzed for their mRNA levels in CRC cells with sh-USP13 or Flag-USP13 expression (Fig. 4A,B) and the mRNA levels of VEGFA, VEGFB, VEGFC, VEGFD, PDGFB, PDGFC, THBS1, WNT7B, PGF, bFGF, MMP2, and CXCL9 were quantified. The mRNA expression levels of VEGFA, VEGFB, VEGFD, PDGFB, PDGFC, THBS1, PGF, and CXCL9 were significantly upregulated in CRC cells with USP13 knockdown, while WNT7B and MMP2 mRNA expression levels were significantly downregulated (Fig. 4A). Overexpression of USP13 downregulated VEGFA, VEGFC, bFGF, and MMP2 of CRC cells (Fig. 4B). The trend in the change of VEGFA expression was consistent with that of USP13 in CRC cells. We subsequently analyzed VEGFA protein expression in CRC cells expressing sh-USP13 or Flag-USP13. The results of ELISA showed that USP13 overexpression remarkably decreased the VEGFA amount of CRC cells, while sh-USP13 markedly increased the VEGFA protein level (Fig. 4C,D). Additionally, the WB assay indicated a negative correlation between USP13 and VEGFA expression levels in CRC cells (Fig. 4E,F). These findings demonstrate the negative regulation of VEGFA expression in CRC cells by USP13.

### 3.4 Tumor Angiogenesis Regulated by USP13 Is Dependent on VEGFA

**Figure 5:**
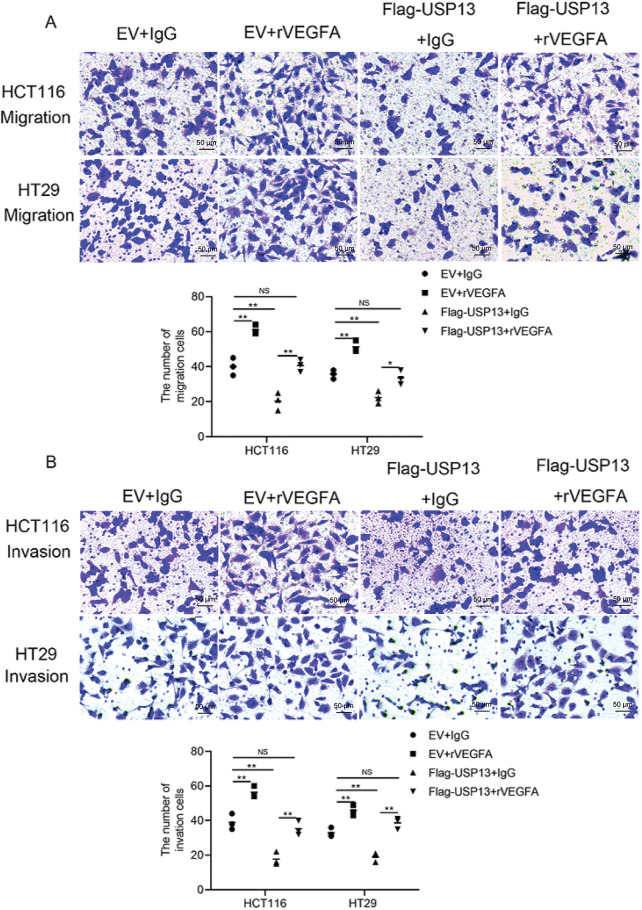
VEGFA contributes to USP13-mediated inhibition of angiogenesis. (**A, B**) HUVEC migration (**A**) and invasion (**B**) after co-treatment with CM in EV cells or Flag-USP13 cells and IgG or rVEGFA were evaluated using Transwell assays. Scale bar: 50 μm. An image of three independent assays is presented. The bar graph depicts HUVEC migration and invasion. Results are represented by mean ± SEM. NS denotes nonsignificant difference; *p < 0.05; **p < 0.01

**Figure 6:**
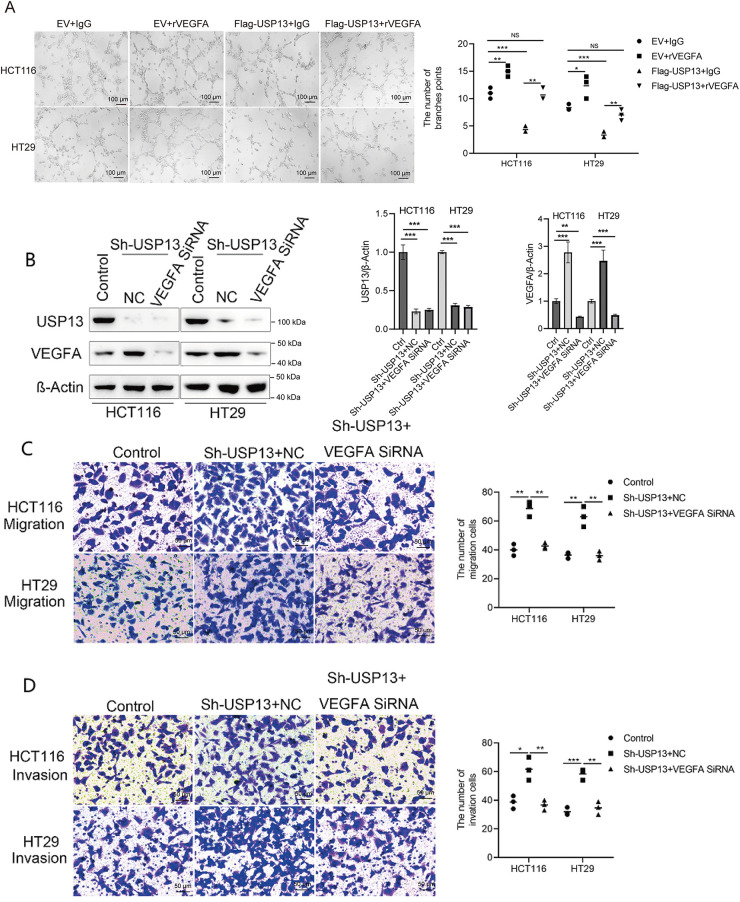
USP13 mediates the inhibition of angiogenesis through VEGFA. (**A**) HUVEC tube formation after co-treatment using CM in EV cells or Flag-USP13 cells and IgG or rVEGFA was investigated. Scale bar: 50 μm. An image from three independent experiments is shown. The bar graph depicts the number of tubes formed by HUVECs. (**B**) WB assay of VEGFA in HCT116 and HT29 cells treated with VEGFA siRNA or NC. β-Actin was used as the loading control. (**C, D**) HUVECs received co-treatment using CM in EV cells or Flag-USP13 cells and IgG or rVEGFA to assess cell migration. A result from three independent assays is displayed. Data are represented by mean ± SEM. NS indicates nonsignificant difference; *p < 0.05; **p < 0.01; ***p < 0.001

Because we observed that USP13 negatively regulates VEGFA expression in CRC and USP13 inversely correlates with MVD, we investigated whether the regulation of HUVEC angiogenesis by USP13 is dependent on VEGFA expression. We found that rVEGFA enhanced HUVEC migration, invasion, and tube formation in comparison with CM from EV-supplemented CRC cells Moreover, USP13 overexpression reduced HUVEC migration, invasion, and tube formation and antagonized the rVEGFA-mediated effects (Figs. 5A,B and 6A). The above findings demonstrate that CM supplemented with rVEGFA effectively counteracts the inhibitory effect of CM from Flag-USP13 CRC cells against HUVEC angiogenesis.

Furthermore, VEGFA-specific siRNA (VEGFA siRNA) significantly reduced VEGFA protein expression of CRC cells (Fig. 6B). HUVEC migration, invasion, and tube formation were markedly enhanced by CM from sh-USP13 CRC cells; however, these properties of HUVECs were inhibited when using CM from sh-USP13 CRC cells after VEGFA siRNA treatment (Fig. 6C,D). These results provide strong evidence that VEGFA is essential for the USP13-mediated angiogenesis-inhibiting effect on HUVECs.

### 3.5 USP13 Inhibits VEGFA and CRC Angiogenesis via the PTEN-AKT Pathway

**Figure 7:**
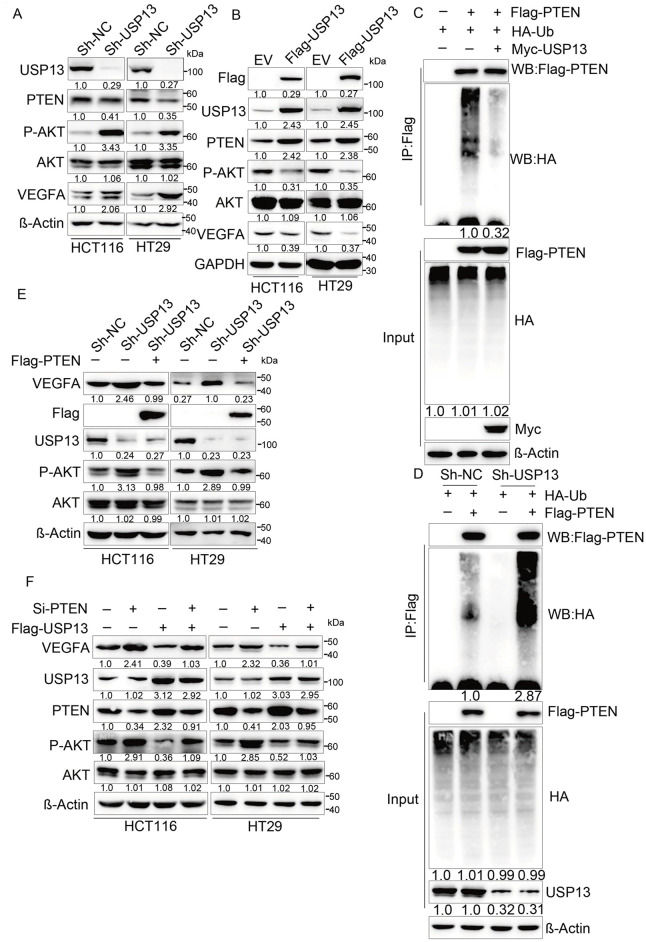
The PTEN-AKT pathway is crucial for exerting the inhibition of USP13 against VEGFA expression and CRC angiogenesis. (**A**) sh-NC and sh-USP13 CRC cells were used in the WB assay to compare USP13, PTEN, and VEGFA levels, with β-Actin being the loading control. (**B**) EV and Flag-USP13 CRC cells were subjected to WB assay to investigate USP13, PTEN, p-AKT, and VEGFA expression. GAPDH was used as the loading control. (**C**) HCT116 cells received co-transfection using MYC-USP13, Flag-PTEN, and HA-ubiquitin (Ub), followed by Flag bead immunoprecipitation and immunoblotting with anti-HA and anti-Flag antibodies. Cells were treated with MG132 (10 µM) for 6 h before harvesting. (**D**) Flag bead immunoprecipitation and immunoblotting with anti-HA and anti-Flag antibodies were performed on HCT116 cells co-transfected with sh-NC, sh-USP13, Flag-PTEN, and HA-ubiquitin (Ub). Cells were treated with 10 µM MG132 for 6 h before collection for analysis. (**E**) WB assay of VEGFA and p-AKT levels of HCT116 and HT29 cells after Flag-PTEN or NC transfection, with β-Actin being the loading control. (**F**) WB assay of VEGFA, PTEN, and p-AKT expression in Flag-USP13 HCT116 and HT29 cells transfected with siRNA-PTEN, with β-Actin being the loading control. Results are represented by mean ± SEM

USP13 is a deubiquitinating enzyme of PTEN, and its absence in breast cancer cells may downregulate PTEN expression, thereby enhancing AKT phosphorylation [16]. Moreover, AKT signaling pathways, including NF-κB, play a role in regulating VEGFA expression [22,23]. Therefore, it was hypothesized that USP13 regulates VEGFA in CRC through the PTEN-AKT pathway. Subsequently, our experiments revealed that USP13 knockdown reduced PTEN protein expression and increased p-AKT levels in HCT116 and HT29 cells (Fig. 7A). Conversely, USP13 overexpression increased PTEN protein expression while decreasing p-AKT levels as compared to those in the EV group (Fig. 7B). The MYC-USP13 group also significantly reduced PTEN ubiquitination in comparison with control group in HCT116 cells (Fig. 7C). In contrast, USP13 knockdown significantly increased the ubiquitination of PTEN (Fig. 7D). Furthermore, PTEN overexpression rescued USP13 knockdown-mediated upregulation and phosphorylation of VEGFA (Fig. 7E). Conversely, PTEN knockdown antagonized VEGFA decrease and p-AKT upregulation induced by USP13 overexpression (Fig. 7F).

**Figure 8:**
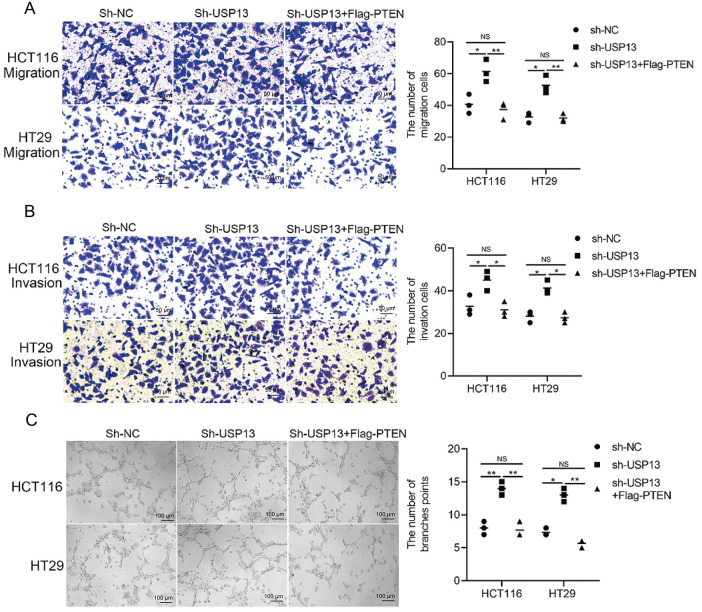
The PTEN-AKT pathway is crucial for USP13 to promote HUVEC migration, invasion, and angiogenesis. (**A, B**) Transwell assays were conducted to evaluate HUVEC migration (**A**) and invasion (**B**) after co-treatment using CM from sh-USP13 cells or sh-USP13 cells with Flag-PTEN. Scale bar: 50 μm. An image of three independent assays is presented. The bar graph displays the number of HUVECs showing migration and invasion properties. (**C**) Tube formation was evaluated in HUVECs after co-treatment using CM derived from sh-USP13 cells or sh-USP13 cells with Flag-PTEN. Scale bar: 50 μm. An image of three independent assays is displayed. The bar graph depicts the number of tubes formed by HUVECs. Results are represented by mean ± SEM. NS indicates nonsignificant difference; *p < 0.05, **p < 0.01

To further elucidate the effect of the USP13-mediated PTEN-AKT signaling pathway on tumor angiogenesis, HUVEC migration, invasion, and tube formation in CM were assessed following the transfection of sh-USP13 CRC cells with Flag-PTEN. As expected, the CM from the sh-USP13 group treated with Flag-PTEN suppressed the migration (Fig. 8A), invasion (Fig. 8B), and tube formation (Fig. 8C) of HUVECs. These findings collectively indicate that USP13 inhibits tumor angiogenesis through the PTEN-AKT signaling pathway.

### 3.6 USP13 Inhibits Tumor Angiogenesis in Nude Mice

**Figure 9:**
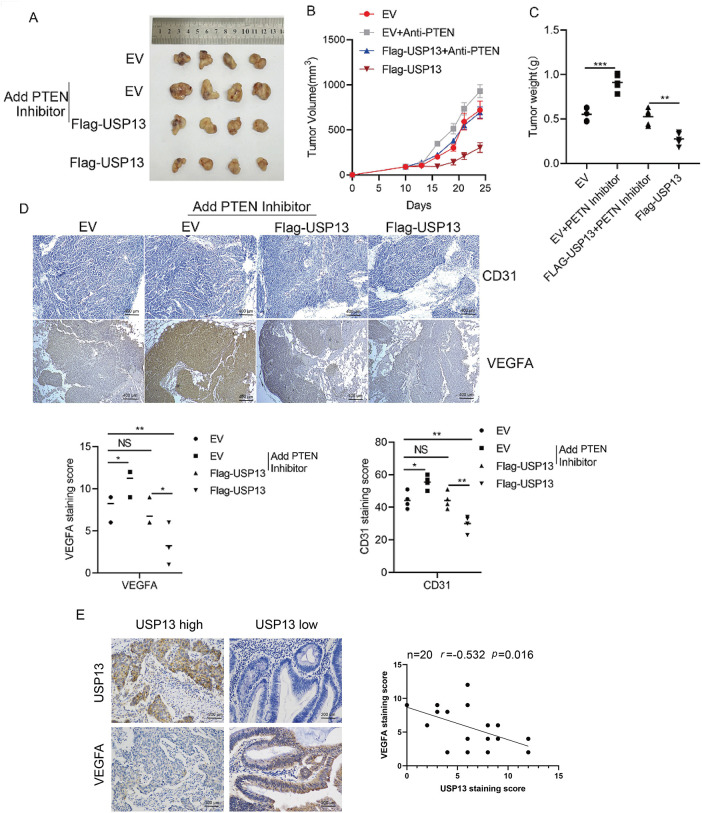
USP13 inhibits angiogenesis through the PTEN/VEGFA pathway in nude mice. (**A**) Subcutaneous tumors were formed by transplantation of EV-HCT116, Flag-USP13-HCT116, EV-HCT116 + PTEN inhibitor (SF1670), and Flag-USP13-HCT116 + PTEN inhibitor (SF1670) cells in nude mice. n = 4. (**B, C**) Statistical analysis of tumor size (**B**) and tumor weight (**C**) of subcutaneous tumors caused by the EV-HCT116, Flag-USP13-HCT116, EV-HCT116 + PTEN inhibitor, and Flag-USP13-HCT116 + PTEN inhibitor groups. n = 4. (**D**) IHC staining index of CD31 and VEGFA protein expression in subcutaneous EV and Flag-USP13 HCT116 tumors as well as EV and Flag-USP13 HCT116 tumors treated with the PTEN inhibitor (3 mg/kg). (**E**) Correlation of USP13 with VEGFA in CRC samples (n = 20). Results are represented by mean ± SEM. NS indicates a nonsignificant difference. *p < 0.05, **p < 0.01. ***p < 0.001

USP13 was analyzed for its effect on tumor angiogenesis of CRC *in vivo*. USP13 overexpression remarkably decreased tumor volume and weight as compared with those in the EV group (Fig. 9A–C). Moreover, PTEN inhibitors mitigated USP13 overexpression-mediated decrease in tumor volume and weight (Fig. 9A–C). Additionally, IHC assay revealed that USP13 overexpression reduced CD31 and VEGFA expression and antagonized PTEN inhibitor-mediated upregulation of CD31 and VEGFA expression in the transplanted CRC tissue (Fig. 9D). These results suggest that USP13 can inhibit CRC angiogenesis by regulating PTEN to suppress VEGFA expression *in vivo*.

Lastly, we analyzed clinical samples to investigate the relationship between USP13 and VEGFA expression. These results suggested that USP13 was negatively correlated with VEGFA in CRC (*p* < 0.05) (Fig. 9E). Therefore, USP13 serves as the critical regulatory factor for angiogenesis inhibition of CRC.

## 4 Discussion

Post-translational modification of proteins is a highly complex regulatory mechanism essential for vital biological functions, including cell growth, differentiation, and apoptosis. Intracellular proteins typically undergo post-translational modifications through processes like ubiquitination, phosphorylation, methylation, and acetylation, resulting in corresponding functional alterations [24]. Ubiquitin regulates not only protein-protein interactions, cellular localization, and substrate-enzyme activity but also participates in proteasome-mediated protein degradation through various ubiquitin linkage sites and other factors. Therefore, protein ubiquitination is a crucial process in organisms [25]. Moreover, protein ubiquitination is inherently unstable and reversible. Specific proteolytic enzymes, also called deubiquitinating enzymes (DUBs), eliminate ubiquitin molecules from proteins. As indicated in current research, DUBs exert an important effect on the ubiquitin-dependent proteasome pathway [26]. Additionally, DUBs regulate the expression and function of numerous other proteins, including tumor suppressors, DNA repair proteins, and epigenetic regulators [27]. USP13, belonging to the DUB family, has an important effect on the oncogenesis of various cancers. As a deubiquitinating enzyme, USP13 exhibits both tumor-promoting and tumor-suppressing effects and is related to the initiation, progression, and apoptosis of breast, bladder, and ovarian cancers and other tumors [12,13,17,28]. To date, the molecular mechanism of USP13 in CRC angiogenesis remains unclear; hence, further investigation is warranted. Here, we hypothesized that USP13 plays an additional significant role in CRC angiogenesis.

Our present research demonstrated that USP13 expression levels are reduced and inversely correlated with CD31 expression levels in CRC patients. This correlation between USP13 and CD31 in tumors has significant implications for our understanding of tumor biology, particularly regarding angiogenesis. CD31 is primarily expressed in endothelial cells and functions as an adhesion molecule that facilitates intercellular signaling and inflammation. The observed correlation between USP13 and CD31 suggests that USP13 may modulate endothelial cell behavior and function, thereby potentially influencing CD31 expression. In this context, investigation of the mechanisms through which USP13 regulates CD31 could reveal novel therapeutic targets for antiangiogenic strategies. Another noteworthy finding is that the CM from sh-USP13 CRC cells stimulated *in vitro* migration, invasion, and tubule formation of HUVECs. Additionally, HCT116 xenografts overexpressing USP13 in nude mice exhibited a significant reduction in MVD. Thus, based on *in vitro* and *in vivo* experimental results, USP13 overexpression of CRC cells inhibits angiogenesis. Therefore, it is postulated that USP13 exerts an essential effect on regulating the angiogenesis of CRC and could thus present a potential avenue for therapeutic intervention.

Tumor angiogenesis can be primarily modulated via various cytokines and growth factors secreted in different cells, including cancer cells, in the tumor microenvironment. In the present work, we examined several VEGFs and related factors for their mRNA expression, like VEGFA, VEGFB, VEGFC, VEGFD, VEGFE, PGF, bFGF, PDGF-BB, WNT7, MMP-2, CXCL-1, and THBS1 [21]. Of them, VEGFA mRNA expression in HCT116 cells exhibited a negative correlation with USP13 expression. We analyzed the VEGFA protein level in CRC cells expressing sh-USP13 or overexpressing USP13 by ELISA. As discovered, VEGFA protein content in CRC cells was the sole factor altered by changes in USP13 gene expression. Therefore, we concluded that USP13 suppresses VEGFA expression in CRC. WB assay revealed elevated VEGFA protein expression levels in sh-USP13-treated CRC cells. Based on these findings, we propose that USP13 inhibits angiogenesis in CRC by suppressing VEGFA expression.

VEGFA is an endothelial cell-specific mitogen that induces both normal and pathological angiogenesis by the activation of several pathways, thereby promoting endothelial cell proliferation, differentiation, migration, and vascular permeability [29]. In CRC, VEGF expression is elevated and correlated with poor prognosis according to previous studies [30]. SIRT2 promotes tumor angiogenesis by regulating STAT3/VEGFA pathway [31]. Elevated levels of VEGF are related to unfavorable prognostic outcomes of CRC patients. Some studies have shown that DHX32 may enhance VEGFA production, thereby promoting angiogenesis in this disease [30,32]. Hence, antiangiogenic therapies for CRC should focus on inhibiting the function of VEGFA in tumor angiogenesis. In the present study, rVEGFA negated the inhibitory effect of the CM from USP13-overexpressing CRC cells against HUVEC angiogenesis, whereas VEGFA siRNA or VEGFA-neutralizing antibodies abolished the inhibitory effect of the CM in sh-USP13 CRC cells against HUVEC angiogenesis. These findings strongly suggest that VEGFA is essential for the critical role of USP13 in regulating CRC angiogenesis.

Although CRC angiogenesis regulation by USP13 is dependent on VEGFA, the precise mechanism through which USP13 modulates VEGFA requires further investigation. Some studies indicate that USP13 inhibits AKT by stabilizing PTEN in bladder cancer, thereby reducing tumor proliferation, invasion, and migration [16]. As a deubiquitinating enzyme of PTEN, USP13 stabilizes PTEN expression in breast cancer [17]. Additionally, PTEN suppresses angiogenic processes and VEGF expression in hepatocellular carcinoma through both phosphatase-dependent and phosphatase-independent pathways [17]. Based on these findings, we propose that USP13 may inhibit VEGFA expression by deubiquitinating and stabilizing PTEN, ultimately reducing tumor angiogenesis in CRC cells. WB assays confirmed that USP13 deubiquitinates PTEN in HCT116 cells. Moreover, in sh-USP13 and Flag-USP13 CRC cells, a positive correlation was observed between USP13 and PTEN expression levels, with AKT phosphorylation levels corresponding to VEGFA expression. Thus, PTEN overexpression in sh-USP13 CRC cells resulted in suppressed VEGFA expression and reduced HUVEC angiogenesis.

**Figure 10:**
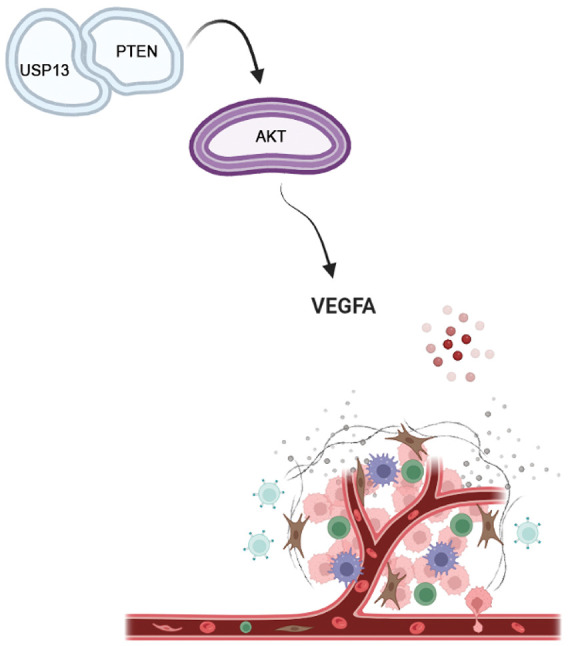
USP13 downregulated VEGFA expression and inhibited angiogenesis through the PTEN-AKT signaling pathway in CRC cells (Drawn by Biorender)

The VEGFA protein expression level in USP13-overexpressing CRC cells was elevated following PTEN siRNA treatment. Moreover, tumor formation experiments in nude mice revealed that tumor cells in the PTEN inhibitor group exhibited accelerated growth and higher MVD in comparison with tumor cells of the control group. These findings substantiate the hypothesis that USP13 inhibits tumor angiogenesis *in vivo* and *in vitro* through the PTEN-AKT-VEGFA axis (Fig. 10).

Despite the significant findings, this study has several limitations. First, the functional analyses primarily relied on HCT116 and HT29 cell lines, which do not fully represent the genetic and molecular heterogeneity of CRC. Additionally, while the PTEN-AKT-VEGFA axis was identified as a key pathway, other potential mechanisms or regulatory factors influencing USP13-mediated angiogenesis remain unexplored. Future studies with larger clinical cohorts, diverse CRC models, and multi-omics approaches are warranted to validate these findings and elucidate the broader regulatory network of USP13 in tumor angiogenesis.

## 5 Conclusion

This study reveals that USP13 suppresses CRC angiogenesis by stabilizing VEGFA through PTEN/AKT signaling. These findings highlight USP13 as a potential therapeutic target for inhibiting tumor vascularization in CRC. Further exploration of USP13’s broader regulatory mechanisms could advance precision therapies for angiogenesis-driven cancers.

## Data Availability

The data that support the findings of this study are available from the corresponding authors upon reasonable request.

## Appendix A

**Table A1:** Primer sequence for RT-qPCR

Human genes	Forward (5^′^–3^′^)	Reverse (5^′^–3^′^)
*VEGFA*	AGGGCAGAATCATCACGAAGT	AGGGTCTCGATTGGATGGCA
*VEGFB*	GAGATGTCCCTGGAAGAACACA	GAGTGGGATGGGTGATGTCAG
*VEGFC*	GAGGAGCAGTTACGGTCTGTG	TCCTTTCCTTAGCTGACACTTGT
*VEGFD*	ATGGACCAGTGAAGCGATCAT	GTTCCTCCAAACTAGAAGCAGC
*PDGFB*	CTCGATCCGCTCCTTTGATGA	CGTTGGTGCGGTCTATGAG
*PDGFC*	GACTCAGGCGGAATCCAACC	CTTGGGCTGTGAATACTTCCATT
*THBS1*	AGACTCCGCATCGCAAAGG	TCACCACGTTGTTGTCAAGGG
*WNT7B*	CACAGAAACTTTCGCAAGTGG	GTACTGGCACTCGTTGATGC
*PGF*	GAACGGCTCGTCAGAGGTG	ACAGTGCAGATTCTCATCGCC
*bFGF*	AGAAGAGCGACCCTCACATCA	CGGTTAGCACACACTCCTTTG
*MMP2*	TACAGGATCATTGGCTACACACC	GGTCACATCGCTCCAGACT
*CXCL9*	CCAGTAGTGAGAAAGGGTCGC	AGGGCTTGGGGCAAATTGTT
*ACTB*	GTTGCTATCCAGGCTGTGCTATCC	GGTGGCAGTGATGGCATGGAC
